# Green Synthesis of Platinum and Palladium Nanoparticles Using *Peganum harmala* L. Seed Alkaloids: Biological and Computational Studies

**DOI:** 10.3390/nano11040965

**Published:** 2021-04-09

**Authors:** Sherif Ashraf Fahmy, Iten M. Fawzy, Basma M. Saleh, Marwa Y. Issa, Udo Bakowsky, Hassan Mohamed El-Said Azzazy

**Affiliations:** 1 Department of Chemistry, School of Sciences & Engineering, The American University in Cairo, AUC Avenue, P.O. Box 74, Cairo 11835, Egypt; sheriffahmy@aucegypt.edu (S.A.F.); basma_saleh@aucegypt.edu (B.M.S.); 2 School of Life and Medical Sciences, University of Hertfordshire Hosted by Global Academic Foundation, R5 New Garden City, New Capital AL109AB, Cairo 11835, Egypt; 3 Pharmaceutical Chemistry Department, Faculty of Pharmaceutical Sciences and Pharmaceutical Industries, Future University in Egypt, Cairo 12311, Egypt; iten.mamdouh@fue.edu.eg; 4 Department of Pharmacognosy, Faculty of Pharmacy, Cairo University, Kasr El-Aini Street, Cairo 11562, Egypt; marwa.issa@pharma.cu.edu.eg; 5 Department of Pharmaceutics and Biopharmaceutics, University of Marburg, Robert-Koch-Str. 4, 35037 Marburg, Germany

**Keywords:** green synthesis, platinum nanoparticles, palladium nanoparticles, *Peganum harmala* seed alkaloid fraction, antioxidant, anticancer

## Abstract

This study reports a facile and eco-friendly method for the green synthesis of platinum and palladium nanoparticles (Pt NPs and Pd NPs) using *Peganum harmala* seed alkaloid fraction. The ζ-potential of the synthesized Pt NPs, Pd NPs and Pt–Pd NPs were −11.2 ± 0.5, −9.7 ±1.2, and −12.7 ± 2.1 mV; respectively. Transmission electron microscopy (TEM) revealed the formation of spherical-shaped nanoparticles with smooth margins. The mean diameters of the synthesized Pt NPs, Pd NPs, and Pt–Pd NPs were determined using TEM analysis and were found to be 20.3 ± 1.9, 22.5 ± 5.7, and 33.5 ± 5.4 nm, respectively. The nanoparticles’ bioreduction was confirmed by ultraviolet–visible (UV–vis) spectroscopy, X-ray diffraction (XRD) and Fourier transform infrared (FTIR) spectroscopy, and their organic contents were determined by thermal gravimetric analysis (TGA). The Pt–Pd NPs mixture showed more pronounced antioxidant activity of 843.0 ± 60 μM Trolox equivalent (TE)/mg NPs compared to the individual Pt NPs (277.3 ± 13.5 μM TE/mg NPs) and Pd NPs (167.6 ± 4.8 μM TE/mg NPs). Furthermore, the Pt–Pd NPs exhibited significant cytotoxic activities against lung cancer (A549) and breast adenocarcinoma (MCF-7) cells, IC_50_ of 8.8 and 3.6 µg/mL, respectively; as compared to Pt NPs (IC_50_ of 10.9 and 6.7 µg/mL, respectively) and Pd NPs (IC_50_ of 31 and 10.8 µg/mL, respectively and compared to carboplatin (IC_50_ of 23 and 9.5 µg/mL, respectively). Moreover, molecular docking studies were conducted to explore the possible anticancer and antioxidant mechanisms of the biogenic nanoparticles. Pt NPs, Pd NPs, and their mixture showed inhibitory activity against cysteine proteinase, which supports their high antitumor activity, but moderate antioxidant activity. In conclusion, Pd-Pt NPs mixture prepared using harmala seed alkaloid fraction showed potential as effective antineoplastic agents.

## 1. Introduction

Recently, noble metals nanoparticles (NNPs), including platinum and palladium nanoparticles (Pt NPs and Pd NPs, respectively), have gained attention as promising tools in cancer therapy and biomedical applications [1,2,3]. Pt NPs and Pd NPs have a localized surface plasmon resonance that produces unique absorption bands in the ultraviolet–visible (UV–Vis) region compared to their bulk counterparts [4,5]. Both nanoparticles can readily be taken up by cancer cells and have been reported to exhibit anticancer and antioxidant activities [6,7,8]. One study reported the synthesis of spherical bimetallic Pt–Pd NPs (10–25 nm) using *Dioscorea bulbifera*. The produced bimetallic NPs demonstrated a significant antineoplastic activity (74%) against human cervical (HeLa) cancer cells compared to individual Pd NPs (33%) and Pt NPs (12.6%) [9].

Various chemical approaches have been used for fabrication of Pt NPs and Pd NPs, however, they commonly use harsh reagents and synthetic capping agents [4,5,10,11]. Thus, biological systems such as microorganisms, seaweeds, and plant extracts have been proposed for simple, safe, and cost-effective fabrication of NNPs [4,5,12]. Plant extracts can be readily prepared and harbor many phytochemicals that could act as natural reductants to reduce platinum (IV) and palladium (II) ions [4,5,13,14,15]. Few studies reported the green synthesis of bimetallic NPs using plant extracts. For instance, spherical gold-palladium NPs (7 nm), were prepared employing *Cacumen platycladi* leaf extract [5]. Luo et al. reported a simple method for green synthesis of Fe–Pd NPs (10–100 nm) using grape leaf extract [5]. A recent study reported the utilization of *Terminalia chebula* aqueous fruit extract for the green synthesis of spherical Ag–Pd NPs with an average particle size of 20 nm zeta potential of −14.4 mV. The bimetallic Ag–Pd NPs showed noticeable anticancer activity against A549 cancer cells with IC_50_ of 48.45 µg/mL [5]. Proteins in plant extracts have different functional groups which coat and stabilize the synthesized NNPs [16]. Most of the reported studies focused on the green synthesis of Au and Ag NPs while few studies addressed the green synthesis of Pt and Pd NPs [4,5]. 

*Peganum harmala* L., belonging to *Zygophyllaceae* family, grows naturally in uncultivated zones in the Middle East and North Africa [17]. The seed of *P. harmala* is enriched in therapeutically active alkaloids, including β-carboline and quinazoline alkaloids (such as harmine, harmane, harmol, harmaline, harmalol, and peganine), jointly known as harmala alkaloids [17]. Also, the seed of *P. harmala* contains oxygenated monoterpenes (such as eugenol), anthraquinones, flavonoids, and polysaccharides which are responsible for the bioreduction of different metal ions [17,18]. The seeds of *P. harmala* possess a broad spectrum of biological molecules which have antimicrobial, antioxidant, and anticancer activities [17,19]. Green synthesis of Ag, Au, and ZnO NPs employing different *P. harmala* extracts has been reported [20,21,22].

**Scheme 1:**
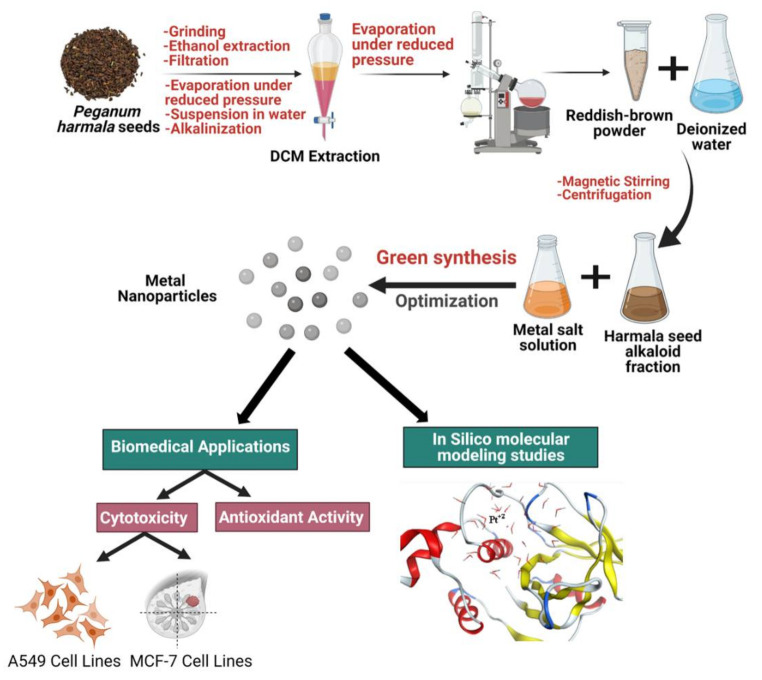
Schematic diagram summarizing the steps involved in the green synthesis of platinum and palladium nanoparticles (Pt NPs, Pd NPs), and Pt–Pd NPs.

In this work, a facile and eco-friendly method was used for green synthesis of Pt NPs, Pd NPs, and Pt–Pd NPs utilizing the alkaloid-rich fraction of *Peganum harmala* seed extract. The greenly synthesized nanoparticles were investigated for cytotoxic and antioxidant activities. In silico molecular modeling studies were conducted to explore their possible modes of action as anticancer and antioxidant agents (Scheme 1).

## 2. Materials and Methods

### 2.1. Materials

#### 2.1.1. Chemicals

Platinum (IV) chloride and palladium (II) acetate were obtained from BLD Pharmatech Co., Limited, Cincinnati, OH, USA. TPTZ (2,4,6-tripyridyl-s-triazine) and FeCl_3_.6H_2_O were purchased from Sigma Aldrich, St. Louis, MO, USA. Streptomycin, penicillin, fetal bovine serum, trichloroacetic acid (TCA), Dulbecco’s Modified Eagle’s Medium (DMEM) for sulforhodamine B (SRB) assay, and Tris(hydroxymethyl)aminomethane were purchased from Lonza, (Basel, Switzerland).

#### 2.1.2. Plant Material

Dried mature seeds of *Peganum harmala* L. were obtained from the local Egyptian market. A voucher specimen was deposited (18.1.17) at the Department of Pharmacognosy Herbarium, Faculty of Pharmacy, Cairo University (Giza, Egypt).

### 2.2. Methods

#### 2.2.1. Preparation of *P. harmala* Seed Alkaloid Fraction

*P. harmala* seeds were ground and then extracted by soaking overnight in 70% ethanol at room temperature. The ethanolic extract was filtered and evaporated under reduced pressure yielding a dark reddish-brown viscous residue which was dissolved in 5% HCl, filtered, and partitioned using dichloromethane. The aqueous layer was adjusted to pH 9 using NH_4_OH and then fractionated with dichloromethane. The dichloromethane layer was washed with water and evaporated under reduced pressure at 40 °C, yielding a reddish-brown powder (*P. harmala* seed alkaloid fraction) which was stored at room temperature in dark glass bottles [17].

#### 2.2.2. Green Synthesis of Platinum and Palladium Nanoparticles (Pt NPs and Pd NPs)

*P. harmala* seed alkaloid fraction powder (0.2 g) was dissolved in 10 mL deionized water by magnetic stirring at 80 °C for 90 min followed by centrifugation at 4 °C and 12,000 rpm for 5 min. The yellow-colored solution was then filtered through Whatman no. 40 filter paper. Ten mL of the solution were added to 90 mL of an aqueous solution containing 1 mM Pt (IV) chloride or 1 mM Pd (II) acetate followed by magnetic stirring for 24 h at 60 °C. Color change from yellow to dark brown indicated the formation of Pt NPs and Pd NPs. A mixture of Pt and Pd NPs was also prepared by adding 10 mL of the alkaloid solution to 90 mL of an aqueous solution containing 1 mM of both Pt (IV) chloride and Pd (II) acetate. The reaction conditions were the same as above. The prepared nanoparticles were collected by centrifugation (12,000 rpm) for 30 min at 4 °C. The pellet was washed with a mixture of ethanol and deionized water and then freeze-dried. 

#### 2.2.3. Characterization of Pt NPs and Pd NPs

UV–vis spectrophotometric tracking for the local surface Plasmon resonance (LSPR) of the greenly synthesized nanoparticles was conducted on a CARY 500 UV–vis–NIR Scan dual-beam spectrophotometer (Varian, Palo Alto, CA, USA). The ζ-potential of the synthesized nanoparticles was studied using a laser Doppler velocimetry employing Zetasizer Nano ZS (Malvern Instruments Herrenberg, Germany) in a clear disposable folded capillary cell (DTS1070, Malvern Instruments). 

The nanoparticles’ morphology and diameter size were examined using transmission electron microscopy (TEM) employing a JEOL-JEM 2100 electron microscope operating at 160 kV. A 50 µL aliquot of the nanoparticles was diluted in the ratio of 1:2 (*v*/*v*) with deionized water and stained with 2% aqueous phosphotungstic acid. This mixture was placed and dried over a carbon-coated copper 200 mesh grid, imagined, and photographed. 

X-ray diffraction (XRD) analysis was performed to study the crystalline and elemental states of the prepared metallic nanoparticles. It was conducted in the range of 5° to 90° (each step is 0.03°, 1.5 s per step) at a generator voltage of 40 kV and current of 30 mA employing the Bruker D8 DISCOVER Family. The functional groups of *P. harmala* seed alkaloid fraction and the green synthesized Pt NPs, Pd NPs, and Pt–Pd NPs were identified using Fourier transform infrared (FTIR) spectroscopy performed on a FTIR- 8400s (Shimadzu, Japan). Samples were first compressed with KBr into disks, scanned, and spectra recorded in the range of 500–4000 cm^−1^. The thermal stability of the nanoparticles was studied using a Q50 TGA Thermogravimetric Analyzer, USA. The lyophilized powder was placed in the platinum pan and heated from 0 to 400 °C at a heating rate of 10 °C/min under a nitrogen atmosphere.

#### 2.2.4. Total Antioxidant Activity

##### Standards and Samples Preparation

Ten serial dilutions (4000, 3000, 2000, 1000, 800, 600, 400, 200, 100, and 50 μM) of Trolox stock solution (positive control; 5 mM in methanol) were prepared. Pt NPs, Pd NPs, and Pt–Pd NPs were prepared at a concentration of 0.68 mg/mL methanol.

##### Ferric-Reducing Antioxidant Power (FRAP) Assay

The ferric-reducing antioxidant power (FRAP) assay relies on the antioxidant’s capability to reduce ferric ions into ferrous ions in the presence of TPTZ forming ferrous-TPTZ complex (intense blue color). The assay was conducted in microplates according to the method described by Benzi et al. with few modifications [23]. Briefly, 190 µL of TPTZ reagent were freshly prepared by mixing 300 mM acetate buffer (pH 3.6), 10 mM TPTZ in 40 mM HCl, and 20 mM FeCl_3_, in a ratio of 10:1:1 *v*/*v*/*v*; respectively. TPTZ was then mixed with 10 µL of the sample in a 96-well plate (*n* = 6) and incubated at room temperature for 30 min in the dark. The resulting blue color was measured using a microplate reader FluoStar Omega (Ortenberg, Germany). The ability of the prepared nanoparticles to reduce ferric ions was expressed as a μM Trolox equivalent (TE)/mg sample using the linear regression equation extracted from the linear dose-response curve of Trolox. Data are represented as means ± standard deviation (SD).

#### 2.2.5. In Vitro Cell Viability Assay

##### Cell Culture

Breast adenocarcinoma cells (MCF-7) and lung cancer cells (A-549) were obtained from the American Type Culture Collection (University Boulevard, Manassas, VA, USA) and maintained in DMEM medium supplemented with streptomycin (100 mg/mL), penicillin (100 units/mL), and 10% heat-inactivated fetal bovine serum. Cells were incubated in 5% (*v*/*v*) CO_2_ at 37 °C.

##### Sulforhodamine B (SRB) Colorimetric Assay

Different concentrations of Pt (IV) ions, Pd (II) ions, Pt–Pd ions, Pt NPs, Pd NPs, and Pt–Pd NPs were added to breast adenocarcinoma cells (MCF-7) and lung cancer cells (A-549) and anticancer activities assessed using SRB assay [24,25,26]. Aliquots of MCF-7 and A-549 cell suspension (5 × 10^3^ cells) were seeded in 96-well plates. Cells were incubated for 24 h at 37 °C and 7% CO_2_ in DMEM medium. Cells were treated with aliquots of 100 μL DMEM containing containing different concentrations of Pt (IV) ions, Pd (II) ions, Pt–Pd ions, Pt NPs, Pd NPs, and Pt–Pd NPs (0.01, 0.03, 0.1, 0.3, 1, 3, 10, 30, 100, and 300 μg/mL). After 72 h, media were discarded, and 10% TCA (150 μL) was added to each well and incubated for 1 h at 4 °C then washed several times with distilled water. The SRB solution (70 μL; 0.4% *w*/*v*) was added to cells and incubated for 10 min in dark at room temperature. Finally, plates were washed with 1% acetic acid (3×) and allowed to dry overnight. TRIS (10 mM, 150 μL) was added to dissolve the protein-bound SRB stain and absorbance measured at 540 nm (FLUOstar Omega, Ortenberg, Germany). The IC_50_ (in µg/mL) was computed from concentration-response curves by Sigma Plot software, version 12.0 (System Software, San Jose, CA, USA), using an E-max model equation. All experiments were conducted in triplicates, and data are demonstrated as mean ± standard deviation.

#### 2.2.6. Computational Studies

Based on the anticancer activities obtained from the in vitro studies, molecular modeling studies were performed to gain an insight into the mechanistic actions of the prepared biogenic nanoparticles as anticancer agents.

Active metals and metal complexes have been widely reported to inhibit several significant enzymes, including thioredoxin reductase (TrxR), cysteine proteinases, kinases, and glutathione transferase, which made them beneficial for cancer treatment [27]. The expression of proteinases in many cancer types is significantly high. Thus they represent vital targets for cancer therapy [28]. Hence, cysteine proteinase was selected as a target for the biogenic nanoparticles molecular docking studies, where its crystal structure was downloaded from pdb; code: 1CV8 [29]. The moderate antioxidant activity of the metal NPs were further investigated by molecular modeling. The crystal structure of superoxide dismutase complexed with Mn^+2^ was downloaded from pdb; code: 6DQY. Minimization and docking procedures were carried out using MOE v.2010, together with dynamic simulations and both 2D and 3D resulted from interactions visualization. Hydrogens were added to downloaded proteins, and “automatically connect and type” was applied for all atoms and bonds. The receptor was selected, and fixing potential was carried out. Then, the sequential unneeded chains and ligands were deleted while pocket and complexed ligands were identified. Although the interaction between nanoparticles and enzyme proteins still lacks the adequate forcefield that could capture such binding, we tried to build a simple model of the three prepared metal nanoparticles (Pd NPs, Pt NPs and Pd-Pt NPs) following similar procedures reported in the literature [30]. The structures of the metals were drawn using the program builder, automatic connect, and type was applied. The dynamic simulation was built using start point zero-till 250 checkpoints, velocity applied, and time 0.5. Only one timing was applied for simulations (0.5 s) as a moderate choice for the simple model. Then, the forcefield applied was set as Rule; Empirical forcefield type, charges were fixed, and the solvent was set as a water droplet to mimic the deionized water previously used in lab work. Solvent molecules were automatically calculated for each nanoparticle type. The protocol used was NPA (Nose–Poincare–Andersen–Hamiltonian equations of motion), where the radius at equilibrium was set to be 11.25, 10.15 and 16.75 nm, respectively, for Pd NPs, Pt NPs and Pd-Pt NPs. Only the equilibrium state was selected out of the produced 250 dynamic conformations for further docking studies to facilitate the visualization of the possible ligand–enzyme interaction at the most stable-least energy state. Enzyme proteins were not subjected to dynamic simulations since the study was based on metal–biomolecule interactions and not on large ligands nor protein–protein interactions; hence, the enzymes remained in fixed constraints. Docking was then computed using placement: Alpha Triangle and rescoring 1: London dG with None refinement, None rescoring 2, and retaining 10 scores.

## 3. Results and Discussion

### 3.1. Ultraviolet–Visible (UV–Vis) Spectroscopy

**Figure 1:**
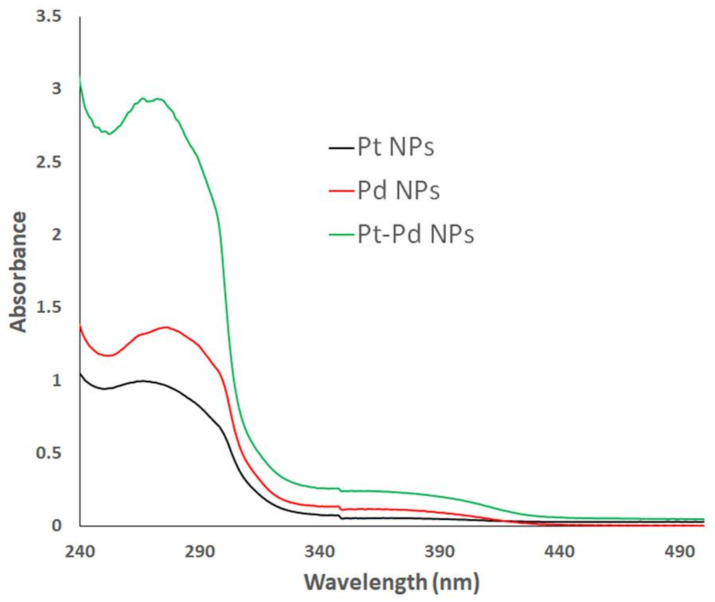
Ultraviolet–visible (UV–Vis) spectra of 0.9 mM Pt NPs, Pd NPs, and Pt–Pd NPs in deionized water synthesized using *P. harmala* seed alkaloid fraction.

The bioreduction of Pt(IV) chloride, Pd(II) acetate, and Pt–Pd salt solutions to biogenic Pt NPs, Pd NPs, and Pt–Pd NPs mediated by *P. harmala* seed alkaloid fraction was tracked visually and using UV–Vis spectroscopy. Initially, the formation of the nanoparticles was confirmed qualitatively by the gradual color change from pale yellow into black, brown, and dark brown colors in the case of Pt NPs, Pd NPs, and Pt–Pd NPs, respectively. The formation of the greenly synthesized NPs was further confirmed by UV–Vis spectroscopy (Figure 1). Significant surface plasmon resonance peaks were observed at 269 and 279 nm for Pt NPs and Pd NPs, respectively. These findings indicated the bioreduction of Pt (IV) and Pd (II) salt solutions into Pt NPs and Pd NPs as reported previously [31,32,33]. The observed absorbance by the Pt–Pd NPs was double that of either individual Pt NPs or Pd NPs.

### 3.2. Transmission Electron Microscopy (TEM) Analysis and ζ-Potential

**Figure 2:**
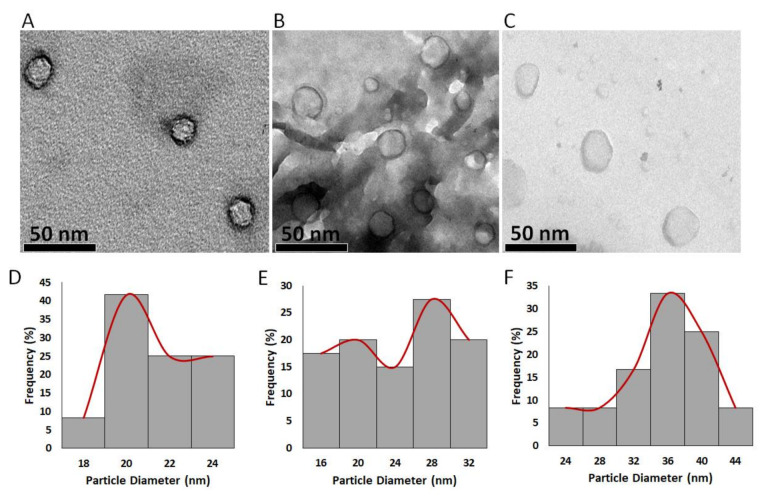
Transmission electron microscopy (TEM) images of (**A**) Pt NPs, (**B**) Pd NPs, and (**C**) Pt–Pd NPs greenly synthesized by *P. harmala* seed alkaloid fraction. Particle diameter (nm) histograms of (**D**) Pt NPs, (**E**) Pd NPs, and (**F**) Pt–Pd NPs created using the image processing program ImageJ (NIH, USA).

Transmission electron microscopy (TEM) analysis was used to determine the particle morphology diameter of the greenly synthesized nanoparticles. As depicted in Figure 2A–C), the particles showed spherical shapes with smooth margins. The mean average diameters of the greenly synthesized Pt NPs, Pd NPs, and Pt–Pd NPs were determined using the image processing program Image J (NIH, USA) and were found to be 20.3 ± 1.9, 22.5 ± 5.7, and 33.5 ± 5.4 nm, respectively. These values lie within the range of the nanoparticles previously reported to passively accumulate into the permeable vasculature of tumor cells via the enhanced permeability and retention (EPR) effect [34,35,36]. 

The ζ-potential of the biogenic Pt NPs, Pd NPs, and Pt–Pd NPs was investigated using laser Doppler velocimetry and demonstrated an outstanding negative surface charge of −11.2 ± 0.5, −9.7 ± 1.2, and −12.7 ± 2.1 mV. The negative charge of nanoparticles’ surface prevents particle aggregation.

### 3.3. X-ray Diffraction (XRD)

**Figure 3:**
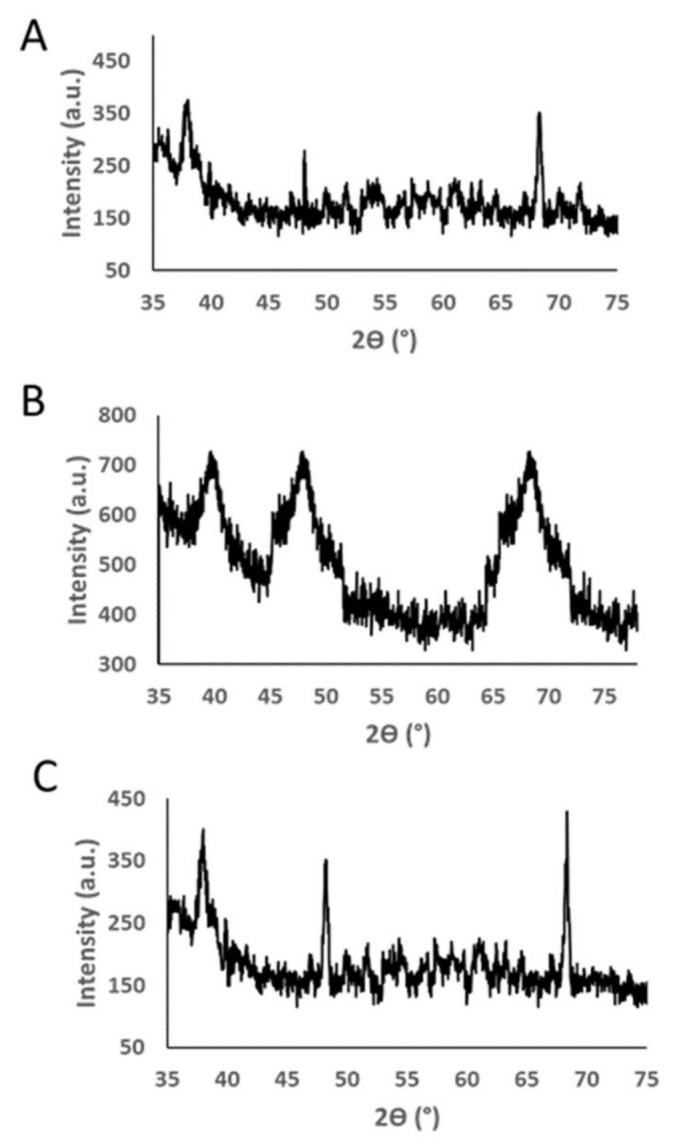
X-ray diffraction (XRD) profiles of (**A**) Pt NPs, (**B**) Pd NPs, and (**C**) Pt–Pd NPs greenly synthesized by *P. harmala* seed alkaloid fraction.

Figure 3 demonstrates the XRD pattern of the greenly synthesized Pt NPs, Pd NPs, and Pt–Pd NPs. Three major diffraction peaks were observed at about 2Ɵ = 38.06°, 48.08° and 68.24° for Pt NPs and 2Ɵ = 39.7°, 48.1°, 68.4° for Pd NPs. These values correspond to the planes (111), (200), and (220) for the face-centered cubic Pt and Pd. The Pt–Pd NPs demonstrated the presence of (111), (200), and (220) peaks, with their corresponding 2Ɵ slightly shifted toward higher values. The XRD data agreed with the previously reported findings for Pt and Pd NPs [16,31,37,38].

### 3.4. Fourier Transform Infrared (FTIR) Spectroscopy and Thermal Gravimetric Analysis (TGA) Analyses

**Figure 4:**
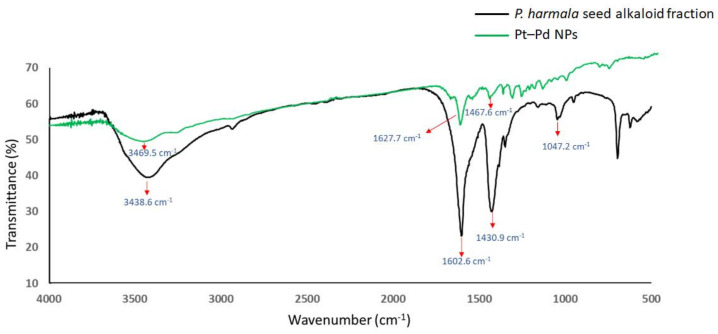
Fourier transform infrared (FTIR) spectra of *P. harmala* seed alkaloid fraction and Pt–Pd NPs.

The FTIR spectra of Pt NPs and Pd NPs were found to be similar to that of the Pt–Pd NPs. Thus, the FT-IR spectra of *P. harmala* seed alkaloid fraction and Pt–Pd NPs were compared (Figure 4). Four major characteristic peaks for *P. harmala* seed alkaloid fraction, were observed at 3438.6 cm^−1^ (–OH stretching), 1602.6 cm^−1^ (C=O asymmetry stretching), 1430.9 cm^−1^ (O–H bending) and 1047.2 cm^−1^ (C–O stretching). The presence of three characteristic peaks of *P. harmala* seed alkaloid fraction in the FTIR spectrum of Pt–Pd nanoparticles may suggest that molecules in the seed extract coated the nanoparticles. However, the peaks’ intensities at 3438.6 cm^−1^ (–OH stretching), 1430.9 cm^−1^ (O–H bending), and 1430.9 cm^−1^ (C–O stretching) were significantly reduced, indicating a lower concentration of *P. harmala* seed alkaloid fraction, possibly indicating the rearrangement and deprotonation of these groups which may be involved in the bioreduction and stabilization processes [34]. Additionally, the peak at 1602.6 cm^−1^ (attributed to the carbonyl group) was significantly diminished in the FTIR spectrum of Pt–Pd NPs, which may suggest conjugation of nanoparticles with the *P. harmala* seed alkaloid fraction via carbonyl groups [16,17,18]. The content of the bioorganic material surrounding the NPs was estimated using thermal gravimetric analysis (TGA) analysis.

**Figure 5:**
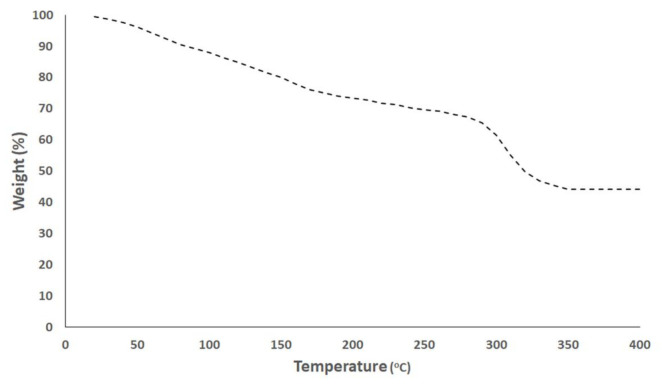
Thermal gravimetric analysis (TGA) thermogram of Pt–Pd NPs synthesized by *P. harmala* seed alkaloid fraction.

The TGA thermogram of Pt–Pd NPs is illustrated in Figure 5 (TGA thermograms of Pt NPs and Pd NPs were found to be similar to that of the Pt–Pd NPs). A constant weight loss of the Pt–Pd NPs was detected from 20 to 400 °C. The thermal decomposition of the bioorganic compounds of *P. harmala* seed alkaloid fraction coating the Pt–Pd NPs reduced their weight by 44.24%. 

### 3.5. FRAP Assay

**Figure 6:**
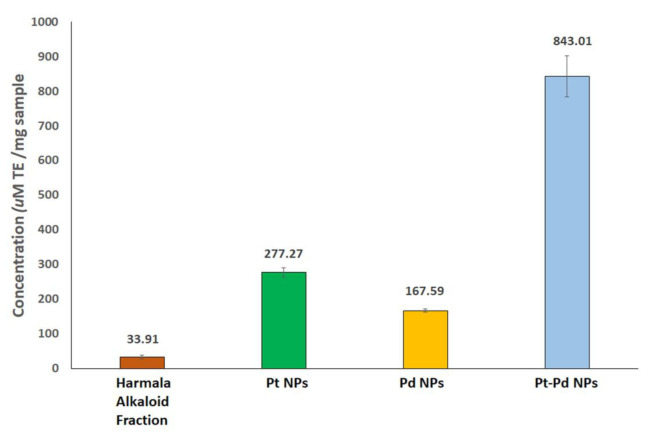
Ferric-reducing antioxidant power (FRAP) assay values for Pt NPs, Pd NPs, and Pt–PdNPs synthesized by *P. harmala* seed alkaloid fraction compared to alkaloid-rich fraction of harmala seed extract (adjusted to the content of the organic material in the NPs, 44.24%). Antioxidant activity is expressed with reference to Trolox standard as μM Trolox equivalent (TE)/mg samples. All trials were conducted in triplicates, and data are presented as mean ± standard deviation.

The antioxidant activities of the prepared metallic nanoparticles compared to harmala alkaloid fraction (adjusted to the content of the organic material in the NPs, 44.24%) were assessed by FRAP assay. The results were expressed with reference to Trolox standard as μM Trolox equivalent (TE)/mg samples. The FRAP values of harmala alkaloid fraction, Pt NPs, Pd NPs, and Pt–Pd NPs were 33.91 ± 5.82, 277.3 ± 13.5, 167.6 ± 4.8, and 843 ± 60 μM TE/mg NPs, respectively, (Figure 6). Notably, Pt (IV), Pd (II), and Pt–Pd ions showed undetectable antioxidant activities. These findings showed that the metal ions’ bioreduction using *P. harmala* seed alkaloid fraction has almost quadrupled their antioxidant activities. Our findings are in good agreement with previous studies that reported remarkable antioxidant activity of the Pt–Pd NPs [39].

### 3.6. In Vitro Cell Viability Assay

The cytotoxic activities of Pt NPs, Pd NPs, and Pt–Pd NPs compared to the individual metals ions were evaluated on lung cancer cells (A549) and breast adenocarcinoma cells (MCF-7) employing SRB assay. The green synthesized metallic nanoparticles exhibited significant in vitro cytotoxic activities compared to the individual metal ions. The cytotoxic activities (IC_50_ in μg/mL, computed by Sigma plot) of the biogenic metallic nanoparticles against both cell lines are presented in Table 1 and Figure S1. The synthesized Pt–Pd NPs revealed the highest cytotoxic activity against A549 and MCF-7 cells, IC_50_ of 8.8 and 3.6 µg/mL, respectively; compared to either Pt NPs (IC_50_ of 10.9 and 6.7 µg/mL, respectively) and Pd NPs (IC_50_ of 31 and 10.8 µg/mL, respectively). This is attributed to the synergistic effect of both transition metals. The synthesized Pt NPs showed higher cytotoxic effects against both A549 and MCF-7 cancer cell lines than Pd NPs. Previous studies showed that the biogenic Pt NPs exert their cytotoxic effects via the induction of apoptosis and cell cycle arrest [40]. Additionally, Pt complexes are used in the design of many platinum-based anticancer drugs, some of which are U. S. Food and Drug Administration (FDA)approved (such as cisplatin, carboplatin, and oxaliplatin); others are still not granted FDA approval (such as nedaplatin) [41]. Moreover, the synthesized Pt–Pd NPs showed higher cytotoxic activity against A549 and MCF-7 cells, when compared to the second-generation platinum-based drug carboplatin (IC_50_ of 23 and 9.5 µg/mL, respectively) [25]. Synthetic platinum-based drugs possess many adverse effects ranging from nephrotoxicity to multi-drug resistance, which hinder their clinical applications [42,43]. On the other hand, the green synthesized NNPs are promising candidates towards the design of safer and effective antitumor agents [44]. Some studies reported the safety and biocompatibility of NNPs capped with plant extracts compared to platinum-based anticancer drugs such as cisplatin, carboplatin, and oxaliplatin [4,5]. Pd NPs produced using *Agaricus bisporus* (mushroom) fungi were found to be biocompatible with red blood cells [45]. Also, Pt NPs synthesized utilizing the aqueous extract of the Indian brown seaweed *Padina gymnospora* were found to be biocompatible to red blood cells with no detected hemolytic activity [46]. Pt NPs produced employing leaf extract of *Maytenus royleanus*, were reported to exhibit anticancer activity against A549 cancer cells and low cytotoxicity against normal cells [47]. These findings will revive interest in platinum-based cancer therapeutics.

### 3.7. Molecular Modeling Studies

#### 3.7.1. Molecular Docking Using Cysteine Proteinase

**Figure 7:**
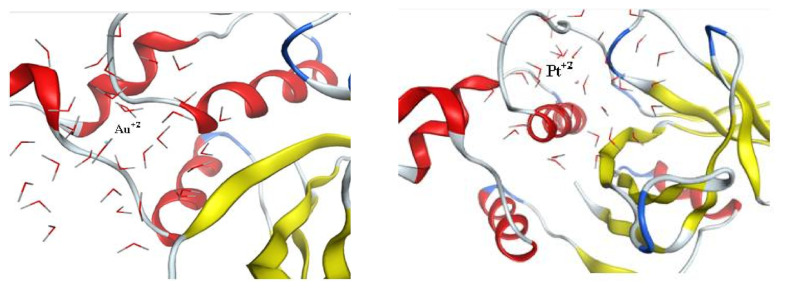
**Left** image: 3D interaction-ligand exposure between Au^+2^NPand key amino acids of cysteine proteinase. **Right** image: 3D interaction-ligand exposure between Pt^+2^NP and key amino acids of cysteine proteinase (both generated by MOEv.2010). The ribbon shape represents the receptor (cysteine proteinase), the lined molecules represents the model surface of the nanoparticles and the solid ball in the middle represents the active metal.

The crystal structure of staphopain cysteine proteinase from *Staphylococcus Aureus* V8 was downloaded from pdb; 1CV8 [29]; its active site was already defined from pdb and was selected as the pocket for interaction in the program software. The metal Au is well known for its inhibitory activity in many cancer types, especially the thiol-containing proteinases [27], thus Au was selected as a reference inhibitor in this molecular docking study and the same dynamic simulation model was applied to Au^+2^ for a fair comparison to the candidate nanoparticles. After preparing the enzyme to be ready for docking, the dynamic nanoparticle models were set using MOEv.2010 and the resulted models were reference (Au^+2^NPs) and tested metals (Pd^0^NPs_,_ Pt^+2^NPs, and Pd^0^-Pt^+2^ mixture). Dynamic models were based on most of the nanoparticles in the reduced forms and only a small percentage in charged forms except for the Pt model where the charged forms constituted most of the model. Molecular docking studies were also performed between the prepared enzymes and each conformer obtained at equilibrium from the prepared four models using MOEv.2010. The results are presented in Table 2. The color map of interactions is supplied in the Supplementary Material (Figure S2) together with the 2D interaction of each tested active metal without the external water surface of the designed nanoparticle (Figure S3). The reference Au^+2^NP displayed a score of interaction energy of the best-docked pose = −17.07 kcal/mol showing ligand exposure to the key amino acid Arg 86, as shown in Figure 7. Although Pd^0^NP showed a higher score than reference = −22.4 kcal/mol, the interaction mode was contrarily presenting ligand exposure to Arg 113. On the other hand, Pt^+2^NP showed both higher score = −26.19 kcal/mol and enhanced mode of interaction with two amino acids ligand exposure; Asn 85 and Arg 86, thus being more embedded in the pocket of the active site as shown in Figure 7. Finally, the Pd^0^-Pt^+2^ mixture displayed the highest score = −30.29 kcal/mol and enhanced interaction similar to that of Pt^+2^NP where Pd^0^ was closer to Asn 85 and Pt^+2^ distanced from Pd^0^ by 6.75 Å, was closer to Arg 86. These findings correlated to those obtained from the in vitro cell viability assays conducted on both cancer cell lines. Thus, in vitro cysteine proteinase inhibition assay may be performed in the future to evaluate the anticancer activities of platinum-based drugs.

#### 3.7.2. Molecular Docking Using Superoxide Dismutase

**Figure 8:**
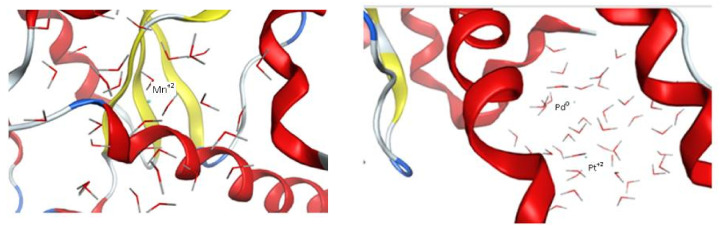
**Left** image: 3D visualization of Mn^+2^NP inside active site pocket of superoxide dismutase. **Right** image: 3D visualization of Pd^0^-Pt^+2^ NPs mixture inside the pocket generated by MOEv.2010. The ribbon shape represented the receptor (superoxide dismutase), the lined molecules represented the model surface of the nanoparticles and the solid ball in middle represented the active metal.

Superoxide dismutase (SOD) protects organisms against potentially damaging oxygen radicals by catalyzing the disproportionation of superoxide to oxygen and hydrogen peroxide [48]. Hence, SOD was selected to explore the prepared biogenic metal nanoparticles effect compared to Mn^+2^ complexed with SOD as a reference. The crystal structure of superoxide dismutase from *Trichoderma reesei* (pdb; 6DQY) was downloaded from the protein data bank. Four dynamic simulated models were built; reference agonist Mn^+2^NPs, Pd^0^NPs, Pt^+2^NPs, and Pd^0^-Pt^+2^ mixture using MOE v.20120. Alpha triangle docking was applied between the prepared superoxide dismutase, and the four built dynamic models. The results (Table 3) show that the reference agonist Mn^+2^NPs with interaction score = −12.37 kcal/mol was embedded in the active pocket site made out of His 30, His 78, Trp 131, Gln 149, Asp 163 and His 167 amino acids without direct ligand exposure as shown in Figure 8. The Pd^0^-Pt^+2^ mixture NPs were similar to that of the reference, only embedded within the same pocket with a score = −10.26 kcal/mol where Pt^+2^ was much closer to the pocket than Pd^0^ that distanced from Pt^+2^ by 6.75 Å. As for Pd^0^ NPs and Pt^+2^ NPs, they displayed different interaction than both reference and the mixture, showing ligand exposure to Ile 207 with docking scores = −11.70 and −13.09 kcal/mol, respectively. The 2D interaction modes of Pd^0^NPs and Pt^+2^NPs are presented in Figure S4. These results also correlated with that of the FRAP assay where the mixture’s antioxidant activity was the best compared to individual NPs.

## 4. Conclusions

This work reports a simple environmentally friendly method for the green synthesis of Pt NPs, Pd NPs, and Pt–Pd NPs employing alkaloid-rich *Peganum harmala* seed extract fraction. The average diameters of the green synthesized spherical Pt NPs, Pd NPs, and Pt–Pd NPs were 20.3 ± 1.9, 22.5 ± 5.7, and 33.5 ± 5.4 nm, respectively. The ζ-potential of the Pt NPs, Pd NPs, and Pt–Pd NPs were −11.2 ± 0.5, −9.7 ± 1.2, and −12.7 ± 2.1 mV, respectively. The metal nanoparticles’ bioreduction was confirmed by UV–vis spectroscopy, XRD, FTIR, and their organic contents determined by TGA analysis. The Pt–Pd NPs showed more pronounced antioxidant activity of 843 ± 60 μM TE/mg NPs compared to the individual Pt NPs (277.3 ± 13.5 μM TE/mg NPs) and Pd NPs (167.6 ± 4.8 μM TE/mg NPs). Furthermore, the Pt–Pd NPs exhibited significant cytotoxic activities against lung cancer and breast adenocarcinoma cells with IC_50_ of 8.8 and 3.6 µg/mL, respectively, compared to the individual Pt NPs (IC_50_ of 10.9 and 6.7 µg/mL, respectively) and Pd NPs (IC_50_ of 31 and 10.8 µg/mL, respectively) and compared to carboplatin (IC_50_ of 23 and 9.5 µg/mL, respectively). The in silico studies were carried out using MOEv.2010, where dynamic simulation models were built up to mimic the prepared nanoparticles’ kinetics. Docking studies showed that Pt and Pd metals exhibited promising activity against the selected cysteine proteinase enzyme and less activity against superoxide dismutase as compared to other metal references under the same conditions. Docking results proposed a possible mechanism of action of the antitumor activity of the biogenic metals, which is supported by the in vitro assay results, via inhibition of a critical proteolytic enzyme. In conclusion, Pt–Pd NPs capped with biological entities from alkaloid-rich harmala seed extract could be used as promising safe antitumor agents. This study will revive interest in Pt-based cancer therapeutics synthesized by green approaches as potentially effective and safe cancer therapy candidates.

## Figures and Tables

**Table 1:** In vitro cytotoxic activities of *P. harmala* seed alkaloid fraction mediated Pt NPs, Pd NPs, and Pt–Pd NPs compared to their individual metal ions against A549 lung cancer cells and breast adenocarcinoma cells (MCF-7).

Cells	In Vitro Cytotoxic Activity (IC_50_ in µg/mL)					
	Pt (IV) Ions	Pd (II) Ions	Pt–Pd Ions	Pt NPs	Pd NPs	Pt–Pd NPs
**A549**	90.8 ± 0.54	91.9 ± 0.67	73.7 ± 0.23	10.9 ± 0.31	31 ± 0.28	8.8 ± 0.11
**MCF-7**	29.8 ± 0.35	48.7 ± 0.73	21.4 ± 0.19	6.7 ± 0.44	10.8 ± 0.59	3.6 ± 0.29

**Table 2:** Molecular docking of the four dynamic metal models against cysteine proteinase enzyme.

Metal Ligand	Score (kcal/mol)	Type of Interaction	Key Amino Acids
**Au^+2^NP (Reference-inhibitor)**	−17.07	Ligand exposure	Arg 86
**Pd^0^NP**	−22.4	Ligand exposure	Arg 113
**Pt^+2^NP**	−26.19	Ligand exposure	Asn 85, Arg 86
**Pd^0^-Pt^+2^NP**	−30.29	Ligand exposure	Asn 85, Arg 86

**Table 3:** Molecular docking of the four dynamic metal models against superoxide dismutase enzyme.

Metal Ligand	Score (kcal/mol)	Type of Interaction	Key Amino Acids
**Mn^+2^NPs** **(Reference-agonist)**	−12.37	Hydrophobic Pocket	His 30, His 78, Trp 131, Gln 149, Asp 163 and His 167
**Pd^0^NPs**	−11.70	Ligand exposure	Ile 207
**Pt^+2^NPs**	−13.09	Ligand exposure	Ile 207
**Pd^0^-Pt^+2^NPs**	−10.26	Hydrophobic Pocket	His 30, His 78, Trp 131, Gln 149, Asp 163 and His 167

## Supplementary Materials

The following are available online at https://www.mdpi.com/2079-4991/11/4/965/s1, Figure S1: Evaluating the cytotoxicity of Pt NPs, Pd NPs, and Pt–Pd NPs compared to the individual metals ions at various concentrations ranging from 0.01 to 300 µg/mL using sulforhodamine B (SRB) assay in (**A**) lung cancer (A549) and (**B**) breast adenocarcinoma (MCF-7) cells. An overall statistically significant decrease in cell viability was observed with the nanoparticles compared to individual metal ions (*p* < 0.05). Untreated cells were used as negative control and considered as 100%. All experiments were carried out in triplicates, and the mean values were calculated. Error bars represent ± standard deviation; Figure S2: Color map of interaction modes between ligands and enzymes provided by MOE v. 2010; Figure S3: 2D interaction-ligand exposure between the four metal dynamic designed nanoparticles (Au^+2^ NPs and tested metals; Pd^0^ NPs, Pt^+2^ NPs and Pd^0^-Pt^+2^ mixture NPs) and key amino acids of cysteine proteinase displayed by MOE v.2010 and Figure S4: 2D interaction-ligand exposure between Pd^0^ NPs and Pt^+2^ NPs designed dynamic models and key amino acids of superoxide dismutase displayed by MOE v.2010.
