# Supplementary material for: Green Synthesis of Platinum and Palladium Nanoparticles Using Peganum harmala L. Seed Alkaloids: Biological and Computational Studies
Source: Nanomaterials (Basel). 2021 Apr 9;11(4):965. doi: 10.3390/nano11040965 (PMC8103518; doi:10.3390/nano11040965)
Supplement: Supplementary file 1 [file nanomaterials-11-00965-s001.zip › SI-nanomaterials-1171620.pdf]

Green Synthesis of Platinum and Palladium Nanoparticles Using *Peganum harmala* L. Seed Alkaloids: Biological and Computational Studies

Sherif Ashraf Fahmy[1,2] , Iten M. Fawzy[3], Basma M. Saleh[1], Marwa Y. Issa[4],Udo Bakowsky*[5], Hassan Mohamed El-Said Azzazy*[1]

[1]Department of Chemistry, School of Sciences & Engineering, The American University in Cairo, AUC Avenue, P.O. Box 74, New Cairo 11835, Egypt

[2]School of Life and Medical Sciences, University of Hertfordshire Hosted By Global Academic Foundation, R5 New Garden City, New Capital AL109AB, Cairo, Egypt

[3]Pharmaceutical Chemistry Department, Faculty of Pharmaceutical Sciences and Pharmaceutical Industries, Future University in Egypt, Cairo 12311, Egypt

[4]Department of Pharmacognosy, Faculty of Pharmacy, Cairo University, Kasr El-Aini Street, Cairo 11562, Egypt

[5]Department of Pharmaceutics and Biopharmaceutics, University of Marburg, Robert-Koch-Str. 4, 35037 Marburg, Germany

Correspondence:

ubakowsky@aol.com (U.B.); hazzazy@aucegypt.edu (H.M.E.-S.A.); Tel.: +49-(0)-6421-28-2-58-84 (U.B.); +2 02 2615 2559 (H.M.E.-S.A.); Fax: +2-02-2795-7565 (H.M.E.-S.A.)

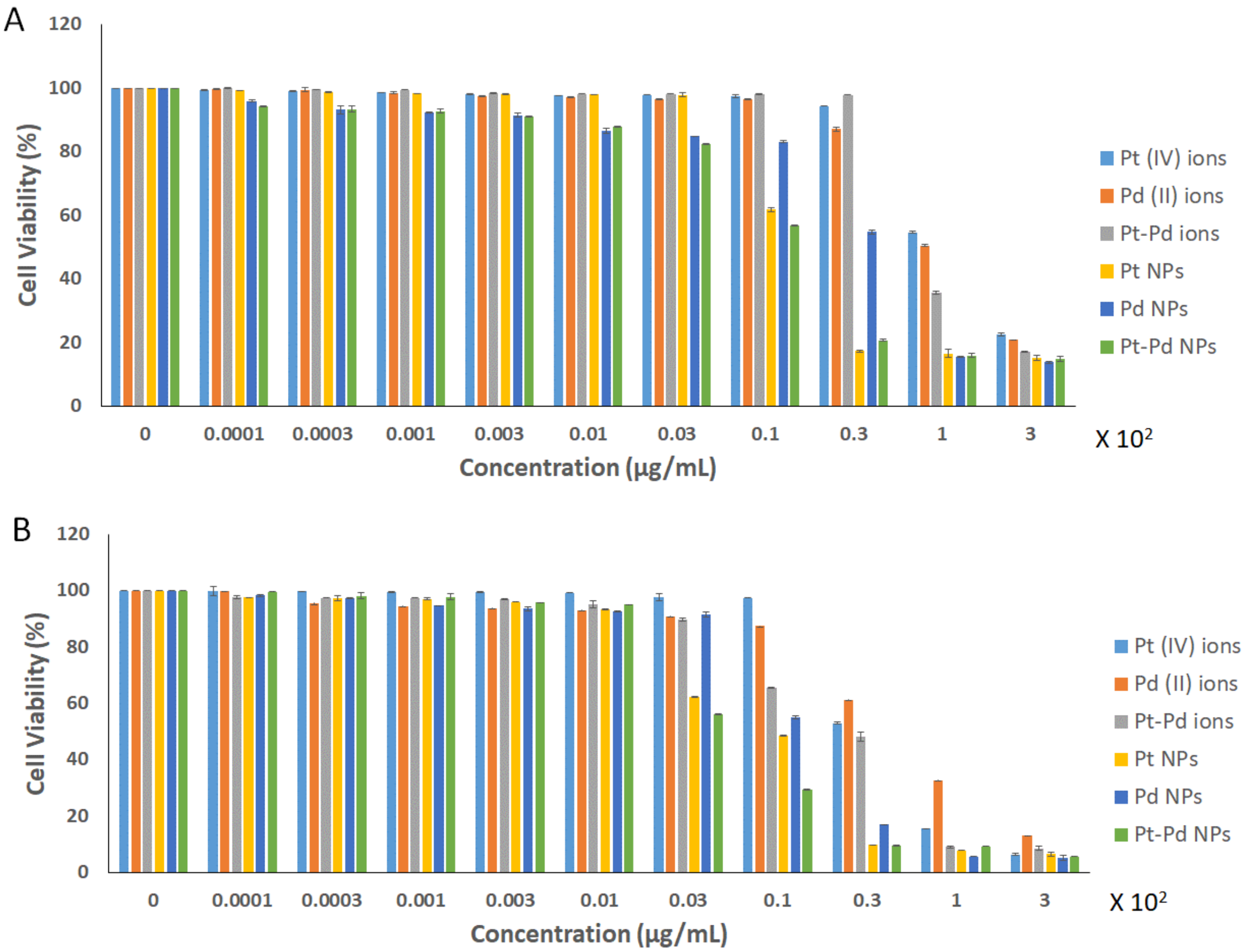

Figure S1. Evaluating the cytotoxicity of Pt NPs, Pd NPs, and Pt-Pd NPs compared to the individual metals ions at various concentrations ranging from 0.01 to 300 µg/mL using SRB assay in (**A**) A549 and (**B**) MCF-7 cells. An overall statistically significant decrease in cell viability was observed with the nanoparticles compared to individual metal ions ($p < 0.05$). Untreated cells were used as negative control and considered as 100%. All experiments were carried out in triplicates, and the mean values were calculated. Error bars represent ± standard deviation.

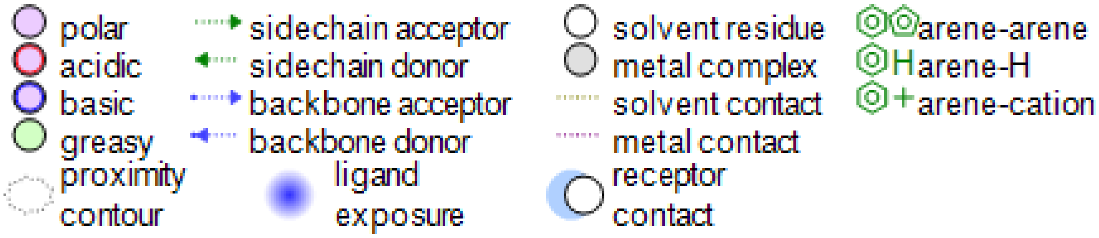

Figure S2. Color map of interaction modes between ligands and enzymes provided by MOE v. 2010.

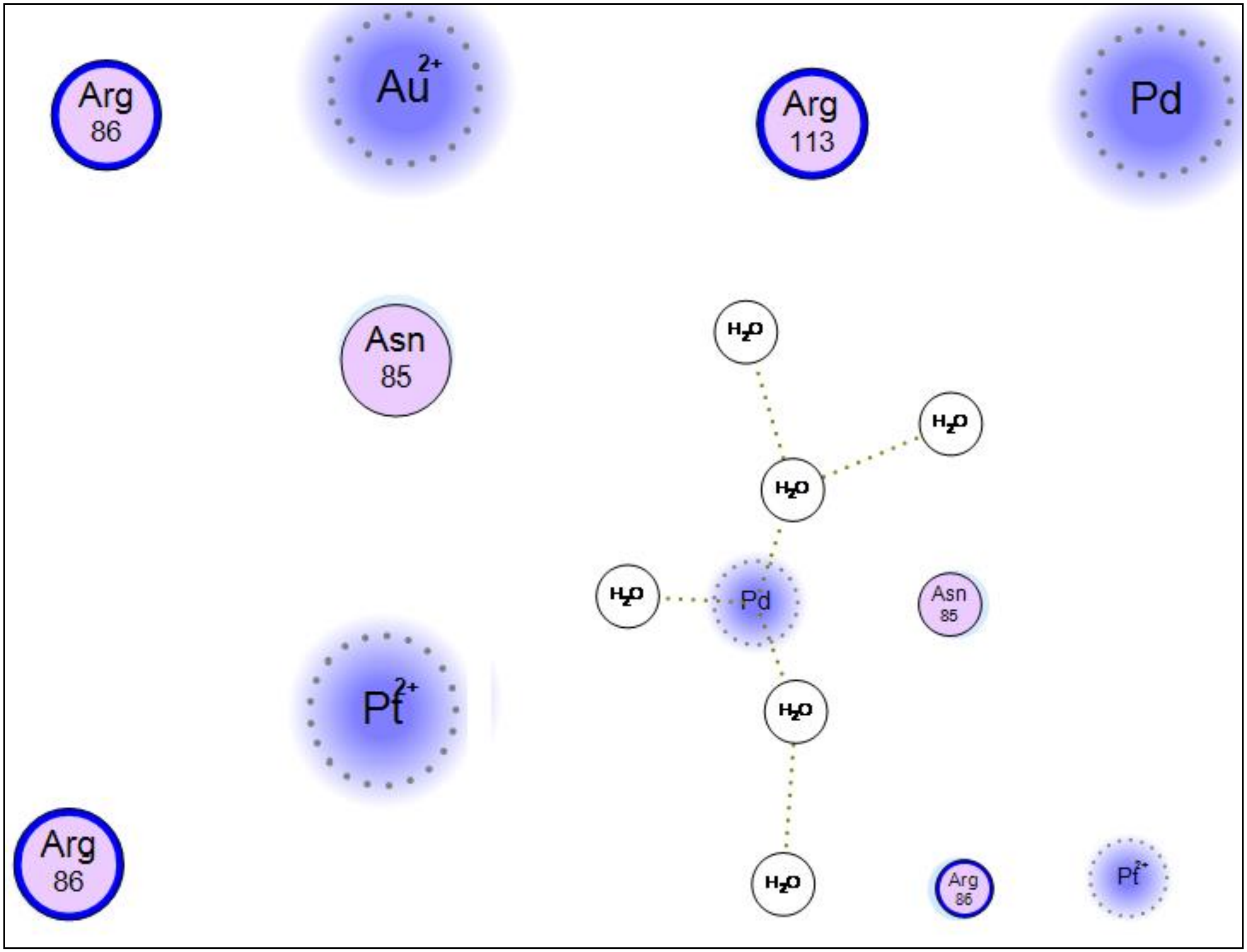

Figure S3:2D interaction-ligand exposure between the four metal dynamic designed nanoparticles (Au^{+2} NPs and tested metals; Pd^0 NPs, Pt^{+2} NPs and Pd^0-Pt^{+2} mixture NPs) and key amino acids of cysteine proteinase displayed by MOE v.2010.

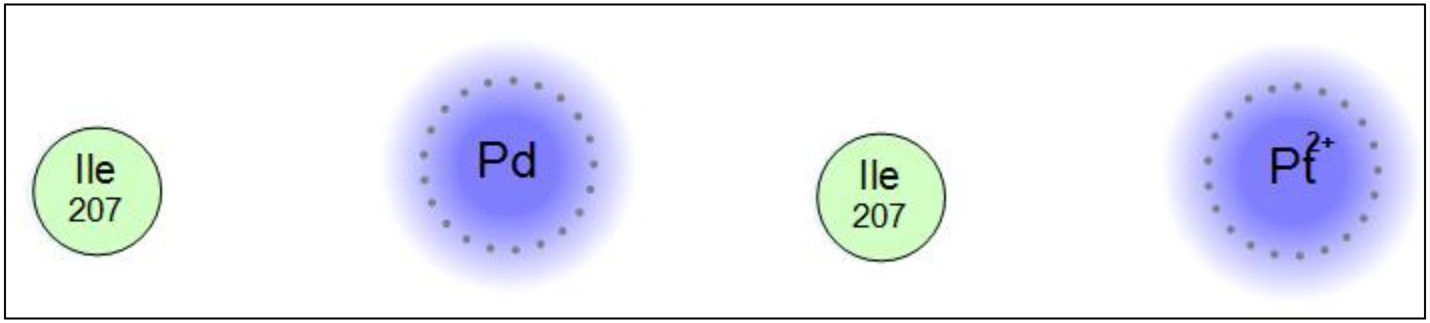

Figure S4:2D interaction-ligand exposure between Pd^0NPs and Pt^{+2} NPs designed dynamic models and key amino acids of superoxide dismutase displayed by MOE v.201
